# APOBEC3 has not left an evolutionary footprint on the HIV-1 Genome

**DOI:** 10.1186/1742-4690-8-S2-P88

**Published:** 2011-10-03

**Authors:** Diako Ebrahimi, Firoz Anwar, Miles P Davenport

**Affiliations:** 1 Centre for Vascular Research, University of New South Wales, Sydney, Australia

## 

It is known that the human immune proteins APOBEC3G and -F (hA3G/F) can inhibit Vif-deficient HIV by G-to-A mutation; however, the roles of these enzymes in the evolution of HIV are debated. We argue that if evolutionary pressure from hA3G/F exists there should be evidence of their imprint on the HIV genome in the form of (i) underrepresentation of hA3G/F target motifs (e.g., TGGG [targeted position is underlined]) and overrepresentation of product motifs (e.g., TAGG) and/or (ii) an increase in the ratio of nonsynonymous to synonymous (NS/S) G-to-A changes among hA3G/F target motifs and a decrease of NS/S A-to-G changes among hA3G/F product motifs. To test the first hypothesis, we studied the representation of hA3G/F target and product motifs in 1,932 complete HIV-1 genomes using Markov models. We found that the highly targeted motifs are not underrepresented and their product motifs are not overrepresented. To test the second hypothesis, we determined the NS/S G7A changes among the hA3G/F target and product motifs in 1,540 complete sets of nine HIV-1 genes. The NS/S changes did not show an increasing/decreasing trend within the target/ product motifs, but the NS/S changes within the motif AG was exceptionally low. We observed the same pattern by analyzing 740 human genes. Given that hA3G/F do not act on the human genome, this suggests a small NS/S change within AG has arisen by other mechanisms. We therefore find no evidence of an evolutionary footprint of hA3G/F. We postulate several mechanisms to explain why the HIV-1 genome does not contain the hA3G/F footprint.

